# Fibroblast Growth Factor 10 Attenuates Renal Damage by Regulating Endoplasmic Reticulum Stress After Ischemia–Reperfusion Injury

**DOI:** 10.3389/fphar.2020.00039

**Published:** 2020-02-07

**Authors:** Xiaohua Tan, Lixia Yu, Ruo Yang, Qianyu Tao, Lijun Xiang, Jian Xiao, Jin-San Zhang

**Affiliations:** ^1^ Department of Pathology, School of Basic Medicine, Qingdao University, Qingdao, China; ^2^ School of Pharmaceutical Sciences, Wenzhou Medical University, Wenzhou, China; ^3^ Department of Pharmacy, Xixi Hospital of Hangzhou, Hangzhou, China; ^4^ Institute of Life Sciences, Wenzhou University, Wenzhou, China

**Keywords:** fibroblast growth factor 10, acute kidney injury, endoplasmic reticulum stress, ischemia–reperfusion, ERK1/2

## Abstract

Renal ischemia–reperfusion (I/R) injury is a predominant cause of acute kidney injury (AKI), the pathologic mechanism of which is highly complex involving reactive oxygen species (ROS) accumulation, inflammatory response, autophagy, apoptosis as well as endoplasmic reticulum (ER) stress. Fibroblast growth factor 10 (FGF10), as a multifunctional growth factor, plays crucial roles in embryonic development, adult homeostasis, and regenerative medicine. Herein, we investigated the molecular pathways underlying the protective effect of FGF10 on renal I/R injury using Sprague–Dawley rats. Results showed that administration of FGF10 not only effectively inhibited I/R-induced activation of Caspase-3 and expression of Bax, but also alleviated I/R evoked expression of ER stress-related proteins in the kidney including CHOP, GRP78, XBP-1, and ATF-4 and ATF-6. The protective effect of FGF10 against apoptosis and ER stress was recapitulated by *in vitro* experiments using oxidative damaged NRK-52E cells induced by tert-Butyl hydroperoxide (TBHP). Significantly, U0126, a selective noncompetitive inhibitor of MAP kinase kinases (MKK), largely abolished the protective role of FGF10. Taken together, both *in vivo* and *in vitro* experiments indicated that FGF10 attenuates I/R-induced renal epithelial apoptosis by suppressing excessive ER stress, which is, at least partially, mediated by the activation of the MEK–ERK1/2 signaling pathway. Therefore, our present study revealed the therapeutic potential of FGF10 on renal I/R injury.

## Introduction

Acute kidney injury (AKI), characterized by rapidly declining glomerular filtration rate (GFR), is a clinical lethal symptom mainly caused by renal ischemia–reperfusion (I/R) injury, sepsis, and nephrotoxic drugs (Mehta et al., 2007; Bonventre and Yang, 2011). AKI is considered as a nosocomial disease with an incidence of 2–7% in hospitalized patients. Despite advances of therapeutic strategies in the past decades, the morbidity and mortality of AKI remain very high (Chertow et al., 2005; Uchino et al., 2005; Bonventre and Yang, 2011; Basile et al., 2012). Renal I/R injury, commonly caused by shock, surgery interventions, kidney transplantation, and toxic insults, accounts for the majority of AKI (Chertow et al., 2005; Ishani et al., 2009). Many previous studies have shown that the pathological mechanism of renal I/R injury is often associated with excessive reactive oxygen species (ROS), oxidative stress, autophagy, inflammation, apoptosis as well as ER stress (Paller et al., 1984; Thadhani et al., 1996; Inagi, 2009; Hotamisligil, 2010; Tan et al., 2017). Although several drugs and therapeutic treatments that could ameliorate renal ischemia injury in animal models have been reported, few of them have been successfully utilized in clinical therapies (Tsuda et al., 2012; Tan et al., 2013). Rapid restoration of renal blood flow after ischemia remains the quickest way to lessen renal tissue damage and functional deterioration caused by ischemia (Paller et al., 1984). However, reperfusion itself also has the potential to elicit additional damage, mainly caused by over-production of ROS, mitochondrial dysfunction, and inflammatory response, which further leads to apoptosis or necrosis (Tsuda et al., 2012; Tan et al., 2013; Inoue et al., 2019). Therefore, effective treatment for AKI is desperately needed.

Endoplasmic reticulum (ER) is a specialized organelle for the synthesis, folding, and trafficking of proteins (Cao and Kaufman, 2012). Many studies have shown that ER is highly sensitive to the changes of the intracellular microenvironment (Cao and Kaufman, 2014; Walter and Ron, 2011). Hypoxia and oxidative stress intrinsic to I/R injury could impair the protein folding of ER. Overaccumulation of unfolded and misfolded proteins triggers the Unfolded Protein Response (UPR) to resolve the excessive ER stress. It has been demonstrated that UPR could expand the ER membrane and thus improve the efficiency of ER for protein folding. UPR could also decrease mRNA translation and reduce protein expression (Schuck et al., 2009). It has been reported that ER stress plays an important role in cell growth, differentiation, and apoptosis. However, excessive activation of ER stress and UPR could activate apoptotic signaling pathways (Hetz et al,. 2012; Tabas and Ron, 2011). Studies have revealed that C/EBP homologous proteins (CHOP), also known as DNA damage inducible transcript 3 (DDIT3), is a master regulator of maladaptive ER stress-induced apoptosis (Rutkowski et al., 2006). Therefore, a strategy focusing on the inhibition of maladaptive ER stress may facilitate the treatment of renal I/R injury.

Fibroblast growth factor 10 (FGF10) is an important member of the FGF family, which mediates mesenchymal to epithelial signaling in a paracrine manner. FGF10 plays a crucial role in embryonic development, wound healing, and tissue regeneration with binding and activating FGF receptor (FGFR) on the cell surface (Itoh and Ohta, 2014). FGF10 highly specifically binds to FGFR2b and initiates the activation of intracellular signaling cascades, including the extracellular signal-regulated kinase (ERK) 1/2 signaling pathway (Zhang et al., 2006; Cho et al., 2009; Wang et al., 2009; Itoh, 2015). Many experimental studies using Fgf10 gene knockout mice have confirmed the crucial role of FGF10 in the development and homeostasis of multiple organs such as the kidney, lung, limb, and pancreatic gland (De Moerlooze et al., 2000; Ohuchi et al., 2000; Beenken and Mohammadi, 2009; Michos et al., 2010; El Agha et al., 2012; Itoh, 2015). It has been reported that FGF10 could accelerate the regeneration of myocardium after myocardial I/R injury (Rochais et al., 2014). Recombinant FGF10 has also been utilized for the treatment of ulcerative colitis and mucositis (Sandborn et al., 2003; Freytes et al., 2004). However, the protective mechanism of FGF10 on renal I/R injury has not yet been fully confirmed. In the present study, we hypothesized that FGF10 could attenuate renal I/R injury by suppressing excessive ER stress and inhibiting renal tubular epithelial cell apoptosis. The protective effect of FGF10 on AKI may be related to the activation of MEK–ERK1/2 signaling pathway. We verified our hypothesis with Sprague–Dawley (SD) rats subjected to renal I/R injury. Rat renal tubular epithelial cell line NRK-52E was also utilized to clarify the protective mechanism of FGF10 in the present study. Results demonstrated that the protective effect of FGF10 on AKI is intimately connected to ER stress which is, at least partially, mediated by the MEK–ERK1/2 signaling pathway.

## Materials and Methods

### Reagents and Antibodies

Bovine serum albumin (BSA), recombinant human FGF10, Tert-Butyl hydroperoxide (TBHP), and U0126 (selective MKK1/2 inhibitor) were purchased from Sigma-Aldrich (St. Louis, MO, USA). Dulbecco's Modified Eagle Medium (DMEM), fetal bovine serum (FBS), Trypsin-EDTA (0.25%), and 4′, 6-Diamidino-2-phenylindole (DAPI) were purchased from Invitrogen (Carlsbad, CA, USA). Antibodies against cleaved Caspase-3 (catalog number: 9661), cleaved Caspase-9 (catalog number: 9507), and phospho-ERK1/2 (catalog number: 9101) were purchased from Cell Signaling Technology, Inc. (Danvers, MA, USA). Anti-ERK1/2 antibody (catalog number: 82380) was purchased from Thermo Fisher Scientific (Sunnyvale, CA, USA). Antibodies against GRP78 (catalog number: ab21685), ATF-6 (catalog number: ab203119), ATF-4 (catalog number: ab23760), PDI (catalog number: ab154820), CHOP (catalog number: ab11419), XBP1 (catalog number: ab37152), and GAPDH (catalog number: ab9485) were purchased from Abcam, Inc. (Cambridge, MA, USA). The secondary antibodies were purchased from Abcam, Inc. (Cambridge, MA, USA) or Santa Cruz Biotechnology, Inc. (Santa Cruz, CA, USA). Annexin V-FITC-PI Apoptosis Detection Kit was purchased from Becton Dickinson, Inc. (San Jose, CA, USA). High sensitivity ECL substrate kit, Hematoxylin and Eosin (H&E) staining kit, and Periodic Acid Schiff (PAS) staining kit were purchased from Abcam, Inc. (Cambridge, MA, USA). The terminal deoxynucleotidyl transferase mediated dUTP nick-end labeling (TUNEL) Assay Kit was purchased form Abcam, Inc. (Cambridge, MA, USA).

### Renal I/R Injury Model and Assessment of Renal Function

To confirm the protective effect of FGF10 treatment on kidney after reperfusion, rat renal I/R injury model was established by surgical operation. Male Sprague–Dawley (SD) rats, eight weeks old, were purchased from Beijing Vital River Laboratory Animal Technology Co., Ltd. and were housed in a Specific-pathogen-free (SPF) facility. The experimental protocol was approved by the Institutional Animal Ethical and Use Committee of Wenzhou Medical University. The rat model of renal I/R injury was established as we described in our previous study (Tan et al., 2017). Briefly, SD rats were anesthetized with intraperitoneal (ip) injection of pentobarbital sodium (25 mg/kg) and placed on a thermostatic surgical table. A small incision was made through the medioventral line and exposed the right kidney. The right kidney was carefully liberated from the surrounding tissue, and nephrectomy was performed. The left kidney was exposed, and the renal artery was clamped using a nontraumatic vascular clamp. Renal blood flow was re-established after 45 min ischemia, and the muscle layer and skin layer were closed using a medical suture. For measurement of renal function, serum creatinine (Cr) was measured 1 day following renal ischemia by the hospital medicine biochemical laboratory (at The First Affiliated Hospital of Wenzhou Medical University). Kidneys were harvested and stored in cryogenic refrigerator for further experiments. Rats were randomly divided into three groups: (a) Sham group: Sham-operated rats with unconstricted renal artery; (b) I/R group: rats were subjected to 45 min of ischemia *via* renal artery followed by reperfusion; (c) I/R–FGF10 group: rats were treated with 0.5 mg/kg FGF10 (ip) 1 h before ischemia. FGF10 was dissolved in sterile saline.

### Cell Culture

The results of *in vivo* experiments in the present study have demonstrated that FGF10 could increase the phosphorylation of ERK1/2 in kidney tissues after reperfusion. To further clarify the role of MEK–ERK1/2 signaling pathway in the protective effect of FGF10, NRK-52E, a rat renal tubular epithelial cell line, was utilized in our present study. We verified the protective effect of FGF10 on damaged NRK-52E induced by TBHP. Furthermore, the participation role of MEK–ERK1/2 signaling pathway in the protective effect of FGF10 was clarified in the *in vitro* experiment. NRK-52E was purchased from the American Type Culture Collection (Manassas, USA) and cultured in DMEM supplemented with 10% FBS, antibiotics (100 units/ml penicillin, 100 μg/ml streptomycin) and incubated under 37°C, 95% air, and 5%CO2. To detect the effect of FGF10 on ER stress induced by TBHP, NRK-52E was cultured on 6-well plates with 2×10^5^ cells per well and randomly divided into four groups: (a) Control group: NRK-52E was cultured in complete medium without any supplement; (b) TBHP group: NRK52E was cultured in complete medium, and then TBHP (200 μmol/L) was added for an additional 12 h; (c) TBHP + FGF10 group: NRK-52Ewas pretreated with recombinant FGF10 (100 ng/ml) for 2 h, and then TBHP (200 μmol/L) was added for an additional 12 h; (d) TBHP + FGF10 + U0126: NRK-52E was pretreated for 2 h with U0126 (20 μmol/L), and then cells were treated the same as TBHP + FGF10 group. The pretreatment compounds in the culture medium were not removed before successive treatment conditions. All experiments with NRK-52E were performed in triplicates.

### Western Blot Analysis

To assess the regulatory role of FGF10 on ER stress and apoptosis, the expression of relevant proteins was analyzed by western blot. For protein analysis of *in vivo* samples, total kidney tissues (contain both of cortex and medulla, but don't contain the renal fibrous capsule) were homogenized and total proteins were extracted using tissue lysis buffer. For protein analysis of *in vitro* samples, NRK-52E cultured in a petri dish was rinsed with PBS buffer three times; total proteins were extracted using cell lysis buffer. An equivalent of 100 μg protein of the *in vivo* sample (30 μg protein of the *in vitro* sample) was separated by Sodium Dodecyl Sulfate PolyAcrylamide and then transferred to a polyvinylidene fluoride (PVDF) membrane for immunoblot analysis. Primary antibodies against cleaved Caspase-3 (1:1,000), cleaved Caspase-9 (1:1,000), Bax (1:3,000), Bcl-2 (1:1,000), GRP78 (1:1,000), CHOP (1:5,000), XBP-1 (1:1,000), ATF-4 (1:1,000), ATF-6 (1:2,000), PDI (1:2,000), ERK1/2 (1:1,000), and phosphor-ERK1/2 (1:1,000) were used in the present study. GAPDH (1:2,500) was used as loading control. The signals were visualized with the ChemiDic™ XRS + Imaging System (Bio-Rad Laboratories). The band densities were quantified with Multi Gauge Software of Science Lab 2006 (FUJIFILM Corporation, Tokyo, Japan).

### Fluorescence Activated Cell Sorting Analysis

To assess the protective effect of FGF10 on NRK-52E against apoptosis induced by TBHP, apoptosis of NRK-52E in each group was quantified with Annexin V-FITC-PI Apoptosis Detection Kit following the manufacturing process instructions. Briefly, NRK-52E was cultured and randomly divided into four groups as described above. Cells were collected and washed twice with PBS and resuspended in binding buffer before the addition of Annexin V-FITC-PI. Cells were then gently vortex mixed and incubated for 15 min in the dark at room temperature before analysis using a BD FACSCalibur™ flow cytometer (BD Biosciences, San Jose, CA, USA) and FlowJo software (Tree Star, San Carlos, CA, USA).

### Immunohistochemistry and Immunofluorescence Staining

To observe the expression and location of ER stress and apoptosis relevant proteins in kidney tissues, immunohistochemistry and immunofluorescence staining were performed. The renal morphology was detected as we described in a previous study (Tan et al., 2017). Briefly, kidneys (both of cortex and medulla) were excised and harvested 1 day after I/R injury. After being dehydrated in gradient ethanol, renal tissue was embedded in paraffin and cut into 5 μm sections. For immunohistochemistry staining, slides were incubated with antibodies against cleaved Capase-3 (1:300), CHOP (1:300), GRP-78 (1:500), and ATF6 (1:300) separately, and then incubated at 4°C overnight. After being incubated with primary antibodies and washed with PBS solution for three times, the slides were incubated with secondary antibodies for 1 h at room temperature, washed with PBS solution for three times, stained with Diaminobenzidine (DAB), and counterstained with hematoxylin. The slides were then subjected to gradient ethanol dehydration, dimethyl benzene transparent, and mounted with neutral resin cover slides. Images were captured using a Nikon ECLPSE 80i. For immunofluorescence staining, slides were incubated with primary antibodies against CHOP (1:300) and GRP-78 (1:500) incubated at 4°C overnight. After reacting with the primary antibodies, the slides were washed 3 times with PBS and then incubated with secondary antibodies (AlexaFluor 488, Abcam) for 1 h at room temperature. Images were captured using a laser confocal microscope (Nikon, Ti-E&A1 plus).

### Renal Histopathology Damage Assessment

To evaluate the renal histopathology damages, slides were stained with hematoxylin and eosin (H&E) and Periodic acid Schiff (PAS), respectively. Each image of the sections was examined under light microscope (Nikon ECLPSE 80i, Japan). Renal histopathology damage degree was evaluated based on intraluminal necrotic cells, cell swelling, interstitial congestion, edema, and protein casts. The following 5 point scoring system was utilized to assess renal damage: 0 point (normal renal morphology), 1 point (damage of kidney tissue ≤10%), 2 points (damage of kidney tissue 11–25%), 3 points (damage of kidney tissue 26–45%), 4 points (damage of kidney tissue 46–75%), 5 points (damage of kidney tissue ≥76%). The pathologists who assessed the images were blinded to the allocation group.

### TUNEL Assay

The Terminal deoxynucleotidyl transferase dUTP nick end labeling (TUNEL) assay is a method for detecting DNA fragmentation. TUNEL has been widely used to identify and quantify cell death in tissues within the last two decades. To assess the protective effect of FGF10 against apoptosis, TUNEL staining and immunohistochemistry staining of cleaved Caspase-3 were used to detect the apoptosis in the kidney tissue after reperfusion. The experimental protocol was exactly the same as we described in our previous study. Kidneys were excised and harvested 1 day after I/R injury. After being dehydrated in gradient ethanol, renal tissue was embedded in paraffin and cut into 5 μm sections. For TUNEL assay, slides were handled with the TUNEL Apoptosis Assay KIT following manufacturing process instructions. The images were captured under a Laser confocal microscope (Nikon, Ti-E&A1 plus). The apoptosis index was analyzed based on five randomly selected images from each group.

### Statistical Analysis

SPSS 19.0 statistical software (Cary, NC, USA) was used for the analysis of rat survival rate after reperfusion. The statistical evaluation of the data was performed using one-way Analysis of Variance (ANOVA) when two groups were compared in this study. Data are expressed as the mean ± SEM of n independent experiments (n ≥ 5). ^*^ *P* < 0.05, ^**^ *P* < 0.01, and ^***^ *P* < 0.001 were considered statistically significant.

## Results

### FGF10 Protects Renal Function and Histological Integrity

**Figure 1:**
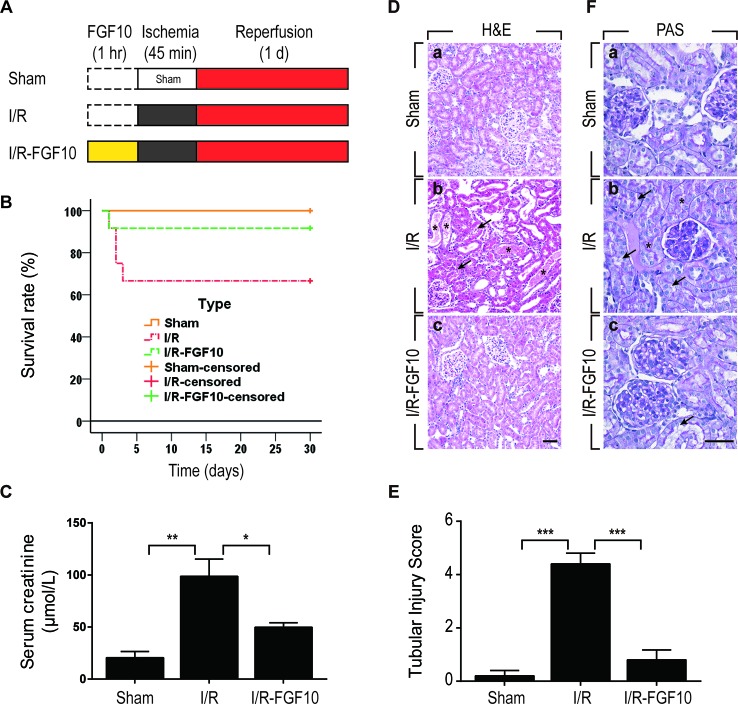
FGF10 attenuates renal I/R injury. **(A)** Flow chart for animal procedures. **(B)** Survival rate. The survival rate of I/R–FGF10 group was significantly improved compared with the I/R group (n = 12). ^*^P < 0.05. **(C)** Serum creatinine levels of animals from Sham group, I/R group and I/R-FGF10 group. ^*^P < 0.05, ^**^P < 0.01. n = 5. **(D)** Histological evaluations of renal tissue stained with H&E staining. Panels are representative of five animals in each group. The arrows point to renal tubular swelling, interstitial congestion and glomerular basement membrane thickening. Asterisks represent protein casts in delated tubulars. Scale bars represent 50 μm. **(E)** Tubular injury score was analyzed based on H&E staining. ^***^P < 0.001. Results are representative of five rats in each group. **(F)** Brush border of renal proximal epithelial cell was evaluated with PAS staining (purple red). The arrows represent the abscission of brush border in proximal tubulars. Asterisks represent tubular lumen narrowing caused by swelling of epithelial cells. Panels are representative of five rats in each group. Scale bars represent 50 μm.

We utilized a rat model of I/R injury to investigate the protective effect of FGF10 on AKI as depicted in **Figure 1A**. To evaluate the protective effect of FGF10, survival rate was analyzed for 30 days after reperfusion. As shown in **Figure 1B**, the 30-days survival rate was significantly improved in the I/R–FGF10 group (91.7%) compared with the I/R group (66.7%). Serum creatinine (Cr) levels were measured at 24 h post reperfusion to assess renal function. As expected, the level of serum Cr was increased nearly five folds in I/R rats (n = 5) compared to the Sham group (**Figure 1C**). However, the level of serum Cr in the I/R–FGF10 group was significantly decreased compared to the I/R rats (P < 0.05). Renal morphological changes were assessed by H&E staining; no obvious damage in the kidney of the Sham group was detected (**Figure 1D-a**), whereas the kidney of the I/R group showed typical pathological features of AKI. The arrows represent intraluminal necrotic cells, swelling of renal tubular cells, interstitial congestion, and edema. The asterisks represent protein casts in delated tubulars (**Figure 1D-b**). Administration of FGF10 significantly attenuated the extent of renal damages (**Figure 1D-c**) and largely preserved the integrity of renal morphology. Tubular injury score was analyzed based on H&E staining. As shown in **Figure 1E**, FGF10 treatment strikingly ameliorated the damage of the kidney tissue after I/R injury. There is no significant difference of the tubular injury between the I/R–FGF10 group and Sham group. PAS staining for glycogen deposition (purple plaques) further indicated that the integrity of brush border on the surface of the renal proximal epithelial cell was damaged in I/R rats. As shown in **Figure 1F-b**, the arrows point to the detachment of brush border from epithelial cell, and the asterisks represent tubular lumen narrowing caused by swelling of epithelial cells. The integrity of the brush border in the I/R–FGF10 group was significantly improved compared with the I/R group (**Figure 1F-c**). In addition, I/R rats displayed significantly increased glycogen accumulation in the glomerulus compared with that of the Sham rats, whereas FGF10 preadministration effectively reduced the deposition of glycogen in glomerulus after reperfusion (**Figure 1F**).

### FGF10 Prevents I/R-Induced Apoptosis of Renal Tubular Epithelial Cells

**Figure 2:**
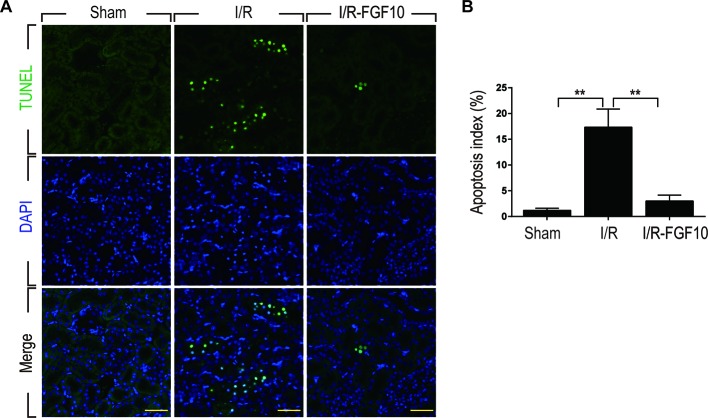
FGF10 reduced cell death in ischemic kidneys. **(A)** Representative sections from kidney tissues 1 day after reperfusion for the detection of nuclear DNA fragmentation performed by terminal deoxynucleotidyltransferase-mediated dUTP nick end labeling (TUNEL) staining. Panels are representative of five rats in each group. Scale bars represent 50 μm. **(B)** Quantitative analysis of the proportion of TUNEL-positive renal tubular epithelial cells in kidney tissues of each group.1.19% in Sham group, 17.3% in I/R group, 1.5% in I/R–FGF10 group. FGF10 significantly reduced the apoptosis of renal tubular epithelial cells after reperfusion. Representative data of five individual samples for each group. ^**^P < 0.001.

TUNEL staining was used to assess the apoptosis of renal tubular cells caused by I/R injury. As shown in **Figure 2A**, few TUNEL-positive cells were observed in the kidney of the Sham group, whereas the number of TUNEL-positive cells in the kidney of the I/R–FGF10 was dramatically increased. Importantly, the number of TUNEL-positive cells in the kidney of the I/R–FGF10 group was markedly reduced compared to the I/R group. Quantification analysis of the number of TUNEL-positive cells revealed that the average percentage of dead cells was 1.19% in the Sham group, 32% in the I/R group, and 4% in the I/R–FGF10 group, respectively (**Figure 2B**).

**Figure 3:**
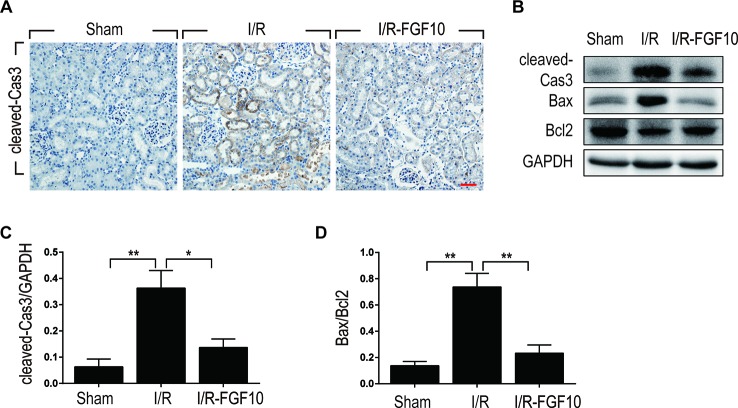
FGF10 reduced the expression of proapoptotic proteins. **(A)** Immunohistochemistry staining of kidney tissues 1 day after reperfusion for the activation of Caspase-3. The expression of cleaved Caspase-3 was significantly increased in the cytoplasm of renal tubular cells after reperfusion, whereas FGF10 treatment reduced the expression of cleaved Caspase-3. Panels are representative of five rats in each group. Scale bars represent 50 μm. **(B)** Western blot analysis of apoptosis index expression. Total kidney tissues (contain both cortex and medulla, but don't contain renal fibrous capsule) were used for the analysis of protein expression in kidney. The expression levels of cleaved Caspase-3, Bax, and Bcl2 were detected. GAPDH was used as a loading control. **(C, D)** The column panels show the normalized optical density analysis. FGF10 significantly reduced the expression of cleaved Caspae-3 and Bax compared with I/R group. ^*^P < 0.05, ^**^P < 0.01.

Caspase-3, also known as CPP32, is synthesized as an inactive proenzyme that is processed to an active form (cleaved Caspase-3) in cells undergoing apoptosis (Fernandes-Alnemri et al., 1994). Previous studies have demonstrated that Caspase-3 is the most important regulatory factor in the apoptotic cell both by death ligand (extrinsic) and mitochondrial (intrinsic) pathways (Salvesen, 2002). The Bcl-2 family, including Bax and Bcl-2, plays a crucial role in the mitochondrial apoptotic pathway (Havasi and Borkan, 2011). To further clarify the mechanism under the protective effect of FGF10 against renal I/R injury, we examined the activation of cleaved Caspase-3 by immunohistochemistry staining. As shown in **Figure 3A**, I/R injury increased the production of cleaved Caspase-3 as demonstrated by strong staining in the cytoplasm of renal tubular cells. The production of cleaved Caspase-3 was markedly decreased in the kidney tissue of the I/R–FGF10 group. Furthermore, several key proteins involved in the regulation of tubular cell apoptosis including cleaved Caspase-3, Bax, and Bcl2 were determined by western blot (**Figure 3B**). As shown in **Figures 3C, D**, the production of cleaved Caspase-3 and Bax in the kidney tissue was markedly increased after reperfusion, whereas administration of FGF10 inhibited the production of cleaved Caspase-3 and Bax. Together, these results indicated that FGF10 preadministration protects the kidneys *via* alleviating apoptosis of the renal tubular epithelial cells after reperfusion.

### The Protective Effect of FGF10 Against Renal I/R Injury Is Associated With ER Stress

**Figure 4:**
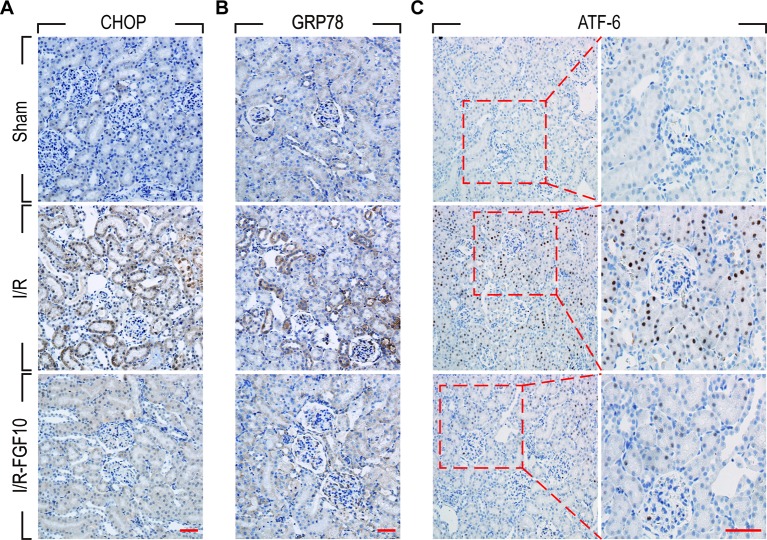
Immunohistochemistry staining of ER stress relevant proteins in kidney tissues after reperfusion. **(A)** Immunohistochemistry staining for CHOP for renal tissues after 1 day of reperfusion. The expression of CHOP was significantly increased in the nucleus and cytoplasm of renal tubular epithelial cells after reperfusion, whereas FGF10 treatment reduced the expression of CHOP. **(B)** Immunohistochemistry staining for GRP78. FGF10 treatment reduced the expression of GRP78 in the cytoplasm of epithelial cells after reperfusion. **(C)** Immunohistochemistry staining for ATF-6. The expression of ATF-6 was significantly increased in the nucleus of renal tubular epithelial cells after reperfusion, whereas FGF10 treatment reduced the expression of ATF-6 compared to I/R alone. Panels are representative of five rats in each group. Scale bars represent 50 μm.

**Figure 5:**
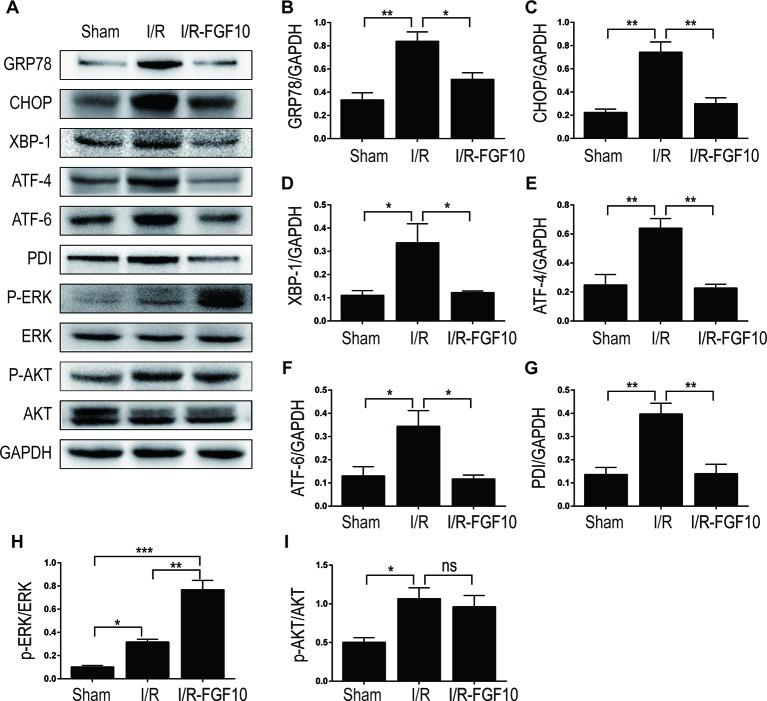
The regulation effect of FGF10 on ER stress and ERK1/2 signaling pathway. **(A)** The expression levels of GRP-78, CHOP, XBP-1, ATF-4, ATF-6, PDI, ERK1/2, and phospho-ERK1/2 in kidney tissues of Sham group, I/R group and I/R-FGF10 group were determined by immunoblot analysis. FGF10 significantly increased the phosphorylation of ERK1/2. **(B–I)** The histograms show the normalized optical density analysis. Results are representative of five rats in each group. ^*^P < 0.05, ^**^P < 0.01, ^***^P < 0.001, *ns* represents no significant difference.

ER stress is a common feature of I/R injury and known to impact renal tubular cell survival. To investigate whether the protective effect of FGF10 on renal tubular cells is associated with the inhibition of excessive ER stress, we examined the expression of ER stress related proteins by immunohistochemistry staining. As shown in **Figures 4A–C**, the expression of CHOP was dramatically increased in the nucleus and cytoplasm of renal tubular epithelial cells after reperfusion, whereas FGF10 treatment reduced the expression of CHOP. FGF10 treatment reduced the expression of GRP78 in the cytoplasm of epithelial cells after reperfusion. The expression of ATF-6 was increased in the nucleus of renal tubular epithelial cells after reperfusion, FGF10 largely reduced the expression of ATF-6 compared to the I/R group. Results of immunohistochemistry staining confirmed that FGF10 could strikingly decreased the expression of ER stress relevant proteins induced by renal I/R injury. Western blotting was used to examine the expression of CHOP, GRP78, ATF-4, ATF6, PDI and XBP1, all of which are ER-stress effectors that, *via* regulation of the unfolded protein response, contribute to cellular homeostasis in kidney. As shown in **Figure 5A** and quantification analysis in **Figures 5B–G**, we observed elevated expression of these proteins in the kidney tissue of the I/R group, whereas pretreatment with FGF10 significantly down-regulated the expression of the proteins mentioned above. These results suggest that preadministration of FGF10 can effectively ameliorate I/R-induced maladaptive ER-stress response, which may contribute to mitigate tubular cell apoptosis. In addition, the apoptosis of renal tubular epithelial cells is the primary reason for AKI caused by I/R injury. We observed that cleaved Caspase-3, CHOP, GRP78, and ATF-6 are mainly expressed in the epithelial cells of the renal tubules (**Figures 3A** and **4**). Based on the results of immunohistochemistry staining, we could infer that FGF10 reduces apoptosis of renal tubular epithelial cells *via* inhibiting the excessive ER stress.

### The Protective Effect of FGF10 Against Apoptosis Is Related to ERK1/2 Pathway

**Figure 6:**
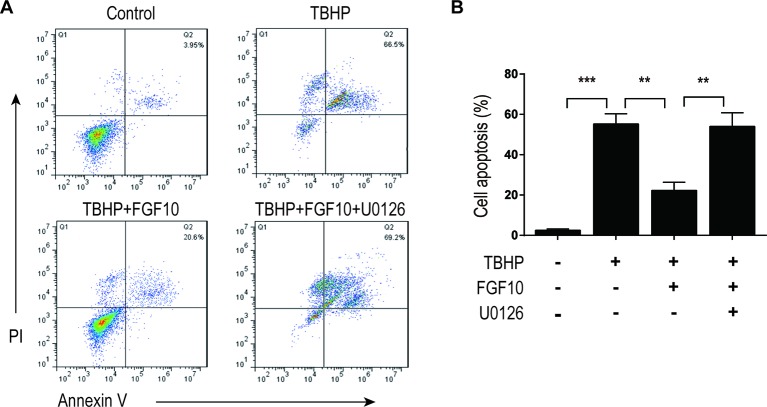
FGF10 inhibits the apoptosis of NRK-52E induced by TBHP. **(A)** Apoptosis of NRK-52E was detected by flow cytometry with annexin V-FITC-/propidium iodide. The top-right panel indicates the apoptotic cells. **(B)** Bar chart represents the apoptosis rate of NRK-52E in each group with three separate experiments. ^**^P < 0.01, ^***^P < 0.001.

**Figure 7:**
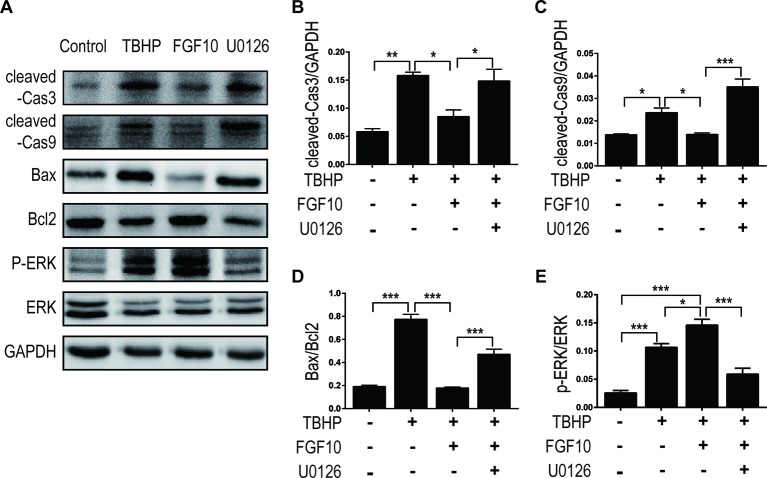
FGF10 reduced the expression of proapoptotic proteins via activating ERK1/2 signaling pathway. **(A)** For protein analysis of *in vitro* samples, total proteins of NRK-52E were extracted using cell lysis buffer. NRK-52E was treated with different culture media and then the expression of cleaved Caspase-3, cleaved Caspase-9, Bax, Bcl-2, ERK1/2, and phosphor-ERK1/2 was detected by western blotting. **(B–E)** Histogram figures show the normalized optical density analysis. Results are representative of five rats in each group. ^*^P < 0.05, ^**^P < 0.01, ^***^P < 0.001.

MAPK/ERK1/2 is a critical downstream pathway of FGF, which plays an important role in the regulation of variety of cellular processes including cell survival, proliferation, migration, and differentiation (Lunn et al., 2007). As mentioned above, FGF10 specifically binds to FGFR2b, which was distributed in the membrane of epithelial cells. To assess the effect of FGF10 on the activation of ERK1/2 pathway, we detected the phosphorylation of ERK1/2 in the kidney tissue of the Sham group, the I/R group, and the I/R–FGF10 group. As shown in **Figures 5A, H**, the phosphorylation of ERK1/2 was mildly increased in the kidney tissue of the I/R group compared to the Sham group. Preadministration of FGF10 led to a robust increase in the phosphorylation of ERK1/2 compared to the I/R group. The PI3K–Akt signal transduction pathway also plays an important role in the regulation of cell survival, proliferation, and migration (Wang et al., 2012). We also detected the phosphorylation of AKT in the kidney tissue of the Sham group, I/R group, and I/R–FGF10 group. As shown in **Figures 5A, I**, the phosphorylation of AKT was increased in the kidney tissue of the I/R group compared to the Sham group. However, there is no significant difference in the phosphorylation of AKT in the kidney tissue between the I/R group and I/R–FGF10 group. Those results may imply that FGF10 protects against renal I/R injury through activating the ERK1/2 signaling pathway, not the PI3K-Akt signaling pathway. To further clarify the relationship between the ERK1/2 signaling pathway and the protective effect of FGF10, we treated NRK-52E cells with TBHP, a commonly ROS inducer which is much stable compared with hydrogen peroxide (H_2_O_2_) solution. The apoptosis of NRK52E cells was detected by flow cytometric analysis with Annexin V-FITC-PI Apoptosis Detection Kit. As shown in **Figures 6A, B**, FGF10 treatment significantly reduced the apoptosis rate of NER-52E caused by TBHP. However, U0126, a selective inhibitor of MEK1/2 that blocks the phosphorylation of ERK1/2 (Shukla et al., 2007), largely abolished the protective effect of FGF10 on NRK-52E cells. To further confirm the role of ERK1/2 signaling pathway in the protective effect against TBHP-induced apoptosis, the expression of cleaved Caspase-3, cleaved Caspase-9, Bax, and Bcl2 was detected by immunoblots. Caspase-9 is an initiator caspase which could further process the activation of other caspases, including Caspase-3, to start the caspase cascade leading to apoptosis. Under the action of the apoptotic signals, the release of Cytochrome c from the mitochondria and activation of Apoptotic protease activating factor 1 (APAF1) cleave pro-caspase 9 into the active form (Li et al., 1997). Our results indicated that FGF10 pretreatment effectively antagonized TBHP-induced Caspase-3 and Caspase-9 cleavages. More importantly, the effect of FGF10 was completely reversed in the presence of MEK inhibitor U0126. Consistently, the drastically increased production of Bax caused by TBHP also appeared to be restored with the treatment of FGF10. However, U0126 exposure partially reversed the effect of FGF10 (**Figures 7A–D**).

As shown in **Figures 7A, E**, FGF10 treatment significantly activated ERK1/2 phosphorylation compared to the TBHP group, which is consistent with what we observed in the kidney tissue. As expected, preaddition of U0126, a highly specific inhibitor of MEK, largely abolished the effect of FGF10 on the phosphorylation of ERK1/2 in NRK-52E cells. These results strongly suggest that ERK1/2 activation is a crucial mechanism in FGF10-mediated protection against cell apoptosis in both I/R injured kidney and TBHP injured NRK-52E cells.

### The Effect of FGF10 on the Regulation of ER Stress Is Related to the Activation of ERK1/2 Pathway

**Figure 8:**
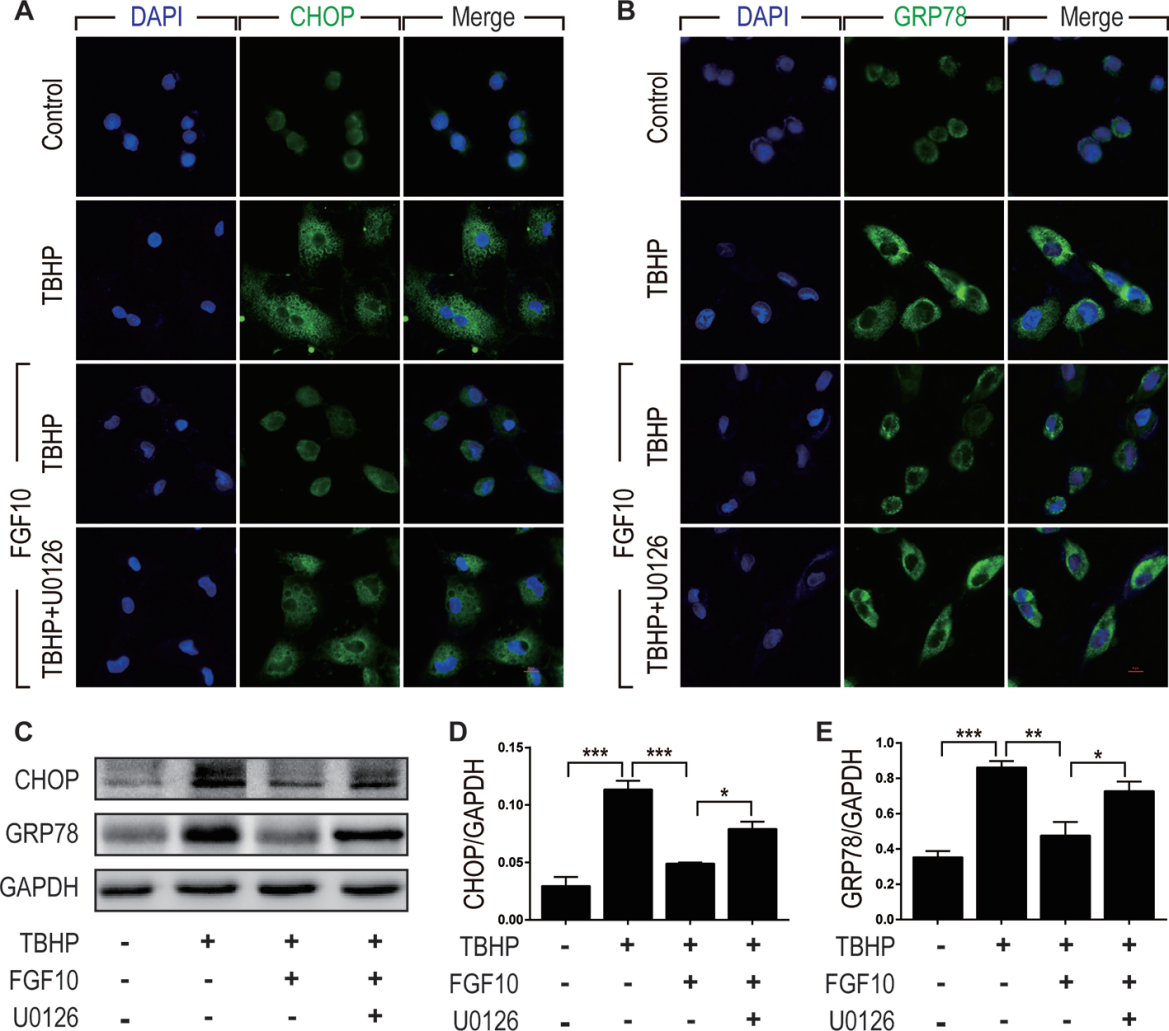
FGF10 attenuates ER stress in NRK-52E cells. **(A, B)** Immunofluorescent staining for CHOP and GRP78 in NRK-52E. FGF10 significantly decreased the expression of CHOP and GRP78 in NRK-52E induced by TBHP, whereas U0126 largely eliminated the effect of FGF10. Panels are representative of five rats in each group. Scale bar represents 50 μm. **(C)** The expression of CHOP and GRP78 was detected by Western blotting. **(D, E)** Bar chart for quantification analysis for the expression of CHOP and GRP78. Results are representative of five rats in each group. GAPDH was used as a protein loading control. ^*^P < 0.05, ^**^P < 0.01, ^***^P < 0.001.

To clarify the relationship between the protective effect of FGF10 and ER stress, we examined the expression of CHOP and GRP78 by immunofluorescence staining in NRK-52E cells respectively (**Figures 8A, B**). We observed that FGF10 effectively attenuated ER stress relevant proteins induced by TBHP, which was inhibited by U0126 to a large extent. The expression levels of CHOP and GRP78 were also examined by western blotting. As shown in **Figures 8C, D, E**, TBHP remarkably increased the expression of CHOP and GRP78 in NRK52E cells, indicating that oxidative stress triggered excessive ER stress in these cells. Similar to the results observed *in vivo*, FGF10 significantly reduced the expression of CHOP and GRP78 in NRK-52E cells treated with TBHP. The effect of FGF10 is partially abolished by U0126. Our results strongly suggested that activation of ERK1/2 signal pathway contributes to FGF10 mediated protection against maladaptive ER stress.

## Discussion

AKI, mainly caused by renal I/R injury, remains a vexing health problem. Despite the current clinical advances in prevention and treatment, the morbidity and mortality of AKI in hospitalized patients remain very high (Basile et al., 2012; Winterberg and Lu, 2012). As a crucial mesenchymal–epithelial signaling growth factor in embryonic development, tissue repair, and regeneration, the role of FGF10 has been investigated in several disease conditions such as cerebral ischemia injury, pulmonary fibrosis, and wound healing (Li et al., 2016; Chen et al., 2017; Chao et al., 2017; El Agha et al., 2017). However, whether FGF10 is capable of delivering a protective effect on AKI in rat model of I/R injury is still unclear.

Currently, many studies reported the relationship between ERK1/2, ER stress, and apoptosis (Sun et al., 2015; Yao et al., 2017). Generally, ERK1/2 are activated upon phosphorylation by MEK1/2 and are considered to promote cell survival (Darling and Cook, 2014). ERK1/2 signaling inhibits apoptosis *via* activating the expression of prosurvival proteins (BCL-2, MCL-1, and BCL-_XL_) and repressing the expression of proapoptotic proteins (BAD, BIM, BMF, and PUMA). However, in some certain conditions such as starvation, ERK1/2 could also promote the expression of NOXA (phorbol-12-myristate-13-acetate-induced protein 1), a proapoptotic member of the BCL-2 family, to decide autophagy or apoptosis (Yao et al., 2017). ER stress could be triggered by a variety of extracellular stimuli and induces apoptosis. It has been reported that renal tubular cell apoptosis induced by I/R injury is associated with excessive ER stress. Excessive ER stress can activate apoptotic signaling pathways *via* CHOP, a master regulator of maladaptive ER stress-induced apoptosis (Rutkowski et al., 2006). The interaction of ERK1/2 signaling pathway and ER stress has been reported in many studies (Zhang et al., 2009; Darling and Cook, 2014). The activation of the ERK1/2 signaling pathway exhibits an antiapoptotic role during ER stress through regulating the IRE1 (inositol requiring enzyme 1) axis of the UPR (Darling and Cook, 2014). FGF10 is a member of the FGF family with multifunctional effect in the regulation of development, wound healing, and tissue regeneration. It has been proved that FGF10 can ameliorate cerebral I/R injury and spinal cord injury *via* inhibiting NF-κB-dependent inflammation and activating the PI3K/Akt signaling pathway (Li et al., 2016; Chen et al., 2017; Dong et al., 2019). In our previous study, we demonstrated that FGF10 can protect the kidney against apoptosis *via* the regulation of inflammatory response and autophagy (Tan et al., 2018). In our present study, the administration of FGF10 can not only reduced the expression of proapoptotic proteins, but also effectively alleviated ER stress kidneys after I/R injury. Therefore, FGF10 exhibits reliable capability in the protection against AKI caused by I/R injury *via* inhibiting maladaptive ER stress.

ER stress and UPR, which could be provoked by glucose depletion and hypoxia after renal I/R injury, have previously shown to play a pivotal role in the enhancement of protein folding ability (Belaidi et al., 2013; Wang et al., 2015). However, excessive and prolonged ER stress and UPR can elicit glomerular and tubular cell damage in patients with AKI and CKD (Bhatt et al., 2008; Inagi, 2009; Xu et al., 2016). Our present results indicated that the expression of ER stress relevant proteins including CHOP, GRP78, XBP-1, ATF-4, ATF-6, and PDI was significantly increased after reperfusion. Importantly, treatment of recombination FGF10 can reduce ER stress relevant proteins and thus inhibited renal tubular cell apoptosis caused by I/R injury. The present study suggested that the renoprotective effect of FGF10 is associated with the regulation of ER stress.

Mitogen-activated protein kinases (MAPKs) are among the most commonly activated signaling pathways associated with various renal injuries (Tian et al., 2000). ERK, an important member of MAPK family, is mainly activated by mitogenic stimuli such as growth factor and hormones. The ERK1/2 signaling pathway is particularly important in the regulation of cell survival, migration, differentiation, and proliferation in a variety of circumstances (Sun et al., 2015; Yao et al., 2017). The role of ERK1/2 in the restoration of renal structure and function is still controversial (Feliers and Kasinath, 2011; Zhang and Cai, 2016; Li et al., 2018). To confirm the role of ERK1/2 in the protective effect of FGF10 on AKI caused by renal I/R injury, we examined the expression of phospho-ERK1/2 in the kidney tissue after reperfusion as shown in **Figure 5**. Our experiments' results confirmed that FGF10 treatment increased the phosphorylation of ERK1/2 in the kidney tissues after reperfusion. As the effect of FGF10 in reducing apoptosis and inhibiting ER stress has been verified in the present study, we speculate that the protective effect of FGF10 in down-regulation of apoptosis may be related to the activation of ERK1/2 signaling pathway.

To further clarify the role of ERK1/2 signaling pathway in the regulation of FGF10 on ER stress after reperfusion, we then examined the protective effect of FGF10 against apoptosis with NRK-52E induced by TBHP. TBHP is a widely used oxidative stress inducer which can increase intracellular ROS production. Our present study demonstrated that FGF10 treatment can strikingly inhibit the apoptosis of NER-52E induced by TBHP. U0126, a specific inhibitor of MKK, abrogated the antiapoptosis effect of FGF10 *via* blocking the phosphorylation of ERK1/2. Moreover, U0126 also reversed the down-regulation effect of FGF10 on ER stress related proteins including GRP78 and CHOP. These results suggest that ERK1/2 signaling pathway is probably the downstream signals induced by FGF10 in the restoration of renal I/R injury. U0126 could suppress the activation of ERK1/2 and abolish the role of FGF10 in the regulation of ER stress on injured NRK-52E induced by TBHP.

As a multifunctional growth factor, FGF10 has been reported to play crucial roles in development and disease. However, the protective mechanism of FGF10 on AKI has not yet been clearly elucidated. In the present study, we confirmed that renal tubular epithelial cell apoptosis induced by hypoxia injury is related to the excessive activation of ER stress. Convincing experimental evidence has been provided both *in vivo* and *in vitro* that exogenously administered FGF10 could attenuate renal tubular epithelial cell apoptosis *via* inhibiting excessive ER stress. Through *in vitro* experiments, we also demonstrated that the protective effect of FGF10 is, at least partly, mediated by MEK–ERK1/2 signaling pathway. In conclusion, results of our present study have implications for understanding the pathophysiology of AKI caused by renal I/R injury and indicate the therapeutic potential of FGF10 in clinical applications. Future research should clarify the exact protective mechanisms of FGF10 in tissue repair and provide novel insights in the field of regenerative medicine.

## Data Availability Statement

All datasets generated for this study are included in the article/supplementary material.

## Ethics Statement

The animal study was reviewed and approved by the Animal Experimentation Ethics Committee of Wenzhou Medical University, Wenzhou, China.

## Author Contributions

XT, JX and J-SZ conceived and designed the experiments. XT, LY, and QT performed the animal operation. XT, LY, RY, and LX performed cell culture, apoptosis assay, FACS analysis, immunoblot, immunohistochemistry, and immunofluorescent staining. XT and LY analyzed the experiments data and prepared the figures. XT and J-SZ wrote and revised the manuscript. J-SZ and JX funded the project.

## Acknowledgments

This study was supported by the National Natural Science Foundation of China (81500519, 81472601) and the Scientific Research Starting Foundation of Qingdao University (DC1900011202).

## Conflict of Interest

The authors declare that the research was conducted in the absence of any commercial or financial relationships that could be construed as a potential conflict of interest
